# Effect of different mixing methods on the bacterial microleakage of white Portland cement and white Mineral Trioxide Aggregate

**DOI:** 10.15171/joddd.2017.016

**Published:** 2017-06-21

**Authors:** Shahriar Shahi, Asmar Bashirzadeh, Hamid Reza Yavari, Farnaz Jafari, Amin Salem Milani, Negin Ghasemi, Mohammad Samiei

**Affiliations:** ^1^Dental and Periodontal Research Center, Faculty of Dentistry, Tabriz University of Medical Sciences, Tabriz, Iran; ^2^Faculty of Dentistry, Tabriz University of Medical Sciences, Tabriz, Iran; ^3^Department of Endodontics, Faculty of Dentistry, Tabriz University of Medical Sciences, Tabriz, Iran; ^4^Department of Endodontics, Dental School, Tabriz Branch, Islamic Azad University, Tabriz, Iran

**Keywords:** Dental cement, dental leakage, *Enterococcus faecalis*, MTA, mixing method

## Abstract

***Background.*** of this study was to investigate the effect of different mixing methods (ultrasonic, amalgamator, and conventional) on the bacterial microleakage of white Portland cement (WPC) and white MTA (Tooth-colored Formula, Dentsply, Tulsa Dental, Tulsa, OK).

***Methods.*** A hundred human single-rooted permanent teeth were decoronated to obtain 14 mm of root length in all the samples. The root canals were cleaned, shaped and obturated. Three millimeters of each root apex were cut off and randomly divided into 6 groups of 15 each (3 groups for WMTA and 3 groups for WPC, each with 3 different mixing methods) and 2 positive and negative control groups (each containing 5 samples). Brain-heart infusion agar (BHI) suspension containing the bacterial species *Enterococcus faecalis* (ATCC 29212) was used for leakage assessment. Statistical analysis was carried out using descriptive statistics and Kaplan Mayer survival analysis with censored data and log rank test using SPSS 18. Statistical significance was set at P<0.05.

***Results.*** The survival means in PC for conventional method, amalgamator, and ultrasonic were 80.2±13.64, 78.5±13.46 and 84.667±11.42 days, with 49.13±12.96, 66±13.32 and 69.07±11.5 days for MTA, respectively. The log rank test showed no significant differences between the three methods in each material (P>0.05).

***Conclusion.*** Bacterial microleakage in the studied samples was not significantly different in terms of the type of the mixing method.

## Introduction

One of the main objectives of root canal treatment is to achieve a proper corono-apical fluid-impervious seal. The presence of microleakage is one of the most important causes for treatment failures.^1-3^ Root-end filling materials are applied to prevent egress of microorganisms and their by-products into periradicular tissue.^4^ Mineral trioxide aggregate (MTA) is widely used in the field of endodontics.^5,6^ In the presence of interstitial fluid, hydration of MTA powder leads to the formation of hydroxyapatite crystals and the formation of a hybrid layer between the dentin and MTA.^7^ MTA has sealing properties and marginal adaptability.^8-11^

Portland cement is similar to MTA in the main compound and contains calcium phosphate, calcium oxide, and silicate.^12^ It has been shown that white and gray Portland cements are more effective in sealing furcal perforations than MTA.^13^ Marginal adaptation of a material with dentin is a critical factor for the success of different endodontic treatments.^14,15^ Studies comparing MTA and Portland cement have shown that these two substances are similar macroscopically and microscopically according to x-ray diffraction.^5,12^

This is also true in the case of endodontic biomaterials such as MTA and PC with regard to the applications of these materials in areas of root canal therapy such as treatment of immature teeth, apical surgery and pulp capping materials; they should have the ability to establish and maintain an appropriate seal.^13,16^ Shahi et al, through two studies on the sealing ability of MTA and PC, demonstrated that the two materials had no differences in dye penetration ability when used as retrograde endodontic materials whereas MTA had more protein leakage compared to PC when applied as a perforation repair material.^13,17^

These materials are hydrophilic cements and the powder-to-liquid ratio and proper mixing influence the physical characteristics of the obtained mixture.^18^ In other words, the mixing technique of these materials influences the hydration degree of the powder particles. Mixing the powder particles with amalgamator can reduce bubbles between the powder particles compared with the manual mixing method, leading to an increase in the wetting of powder particles and homogeneity of the mixture. Ultrasonic mixing of the powder and liquid particles increases powder dispersion. As the reaction surface of the powder particles increases, the hydration levels increase.^18-21^

The research conducted by Basturk et al reflects the increasing compressive strength, hardness and density of MTA with amalgamator and ultrasonic mixing procedures. Additionally, the setting time of the obtained mixture has been reduced by these methods.^19,20^ Shahi et al showed that the mixing technique has no effect on the WT, film thickness, dimensional change, PH, push-out bond strength and compressive strength of MTA; however, it increases the solubility and flow rate and also reduces the setting time.^18,21,22^

Since the effect of mixing method on bacterial leakage of WMTA and PC has not yet been studied, the present study aimed to investigate the effect of different mixing methods on bacterial microleakage of WMTA and PC. The null hypothesis stated that different mixing methods cannot alter bacterial microleakage of PC and WMTA.

## Methods

In this in vitro study, 100 human single-rooted permanent teeth were used. The teeth were decoronated at the cementoenamel junction in order to achieve an identical 14-mm root length in all the samples. The working length was determined at 14 mm. The root canals were prepared with #10, #15, #20 K-files (Dentsply, Maillefer, Ballaigues, Switzerland), followed by RaCe rotary files (FKG, La-Chaux De Fonds, Switzerland) #0.10/40, #0.08/35, #0.06/30 and #0.04/30, using the crown-down technique. MAF size was set at #30.

Root canal irrigation was carried out using 0.5% NaOCl. The smear layer was removed with 10 mL of 2.5% NaOCl and 10 mL of 17% EDTA (Diadent Inc, Chongchong Buk Do, Korea), followed by 5 mL of saline solution as a final irrigation solution. All the teeth were checked again for cracks under a light stereomicroscope (Nikon SMZ 800, Osaka, Japan) at ×40 magnification. Cracked teeth were discarded and replaced.

After drying with sterile paper points (Ariadent, Tehran, Iran), all the teeth were filled with gutta-percha and AH26 sealer (DeTrey, Dentsply, Konstanz, Germany) using lateral compaction obturation technique. The samples were incubated for 72 hours at a temperature of 37^o^C and stored in 100% humidity for 7 days. In the next step, two layers of nail varnish were placed on all the root surfaces except for the apical 2 mm and the coronal plane. In the negative control group, all the root surfaces were covered. Three millimeters of the root end were cut off. After this, 3 mm of root end cavity preparation were performed. The teeth were randomly divided into 6 groups of 15 each: 3 groups for white MTA (Tooth-colored Formula, Dentsply, Tulsa Dental, Tulsa, OK) and 3 groups for WPC, each with 3 mixing methods of ultrasonic, amalgamator, and conventional and 2 positive and negative control groups (each containing 5 samples). In the positive control group, 3 mm of root-end cavity remained empty.

The experimental materials were used according to manufacturer’s instructions and the powder-to-liquid ratio was considered 3:1. Then the specimens were radiographically examined for the length and density of the sealing material. In conventional mixing method, the materials were mixed according to manufacturer’s instructions. In ultrasonic mixing method, the mixing protocol was the same as above except for 20 seconds of using ultrasonic scaler (Juya Electronics, Iran) for this purpose. Liquid and powder forms of the materials were mixed and placed in a amalgamator (Duomate II, Germany) for 20 seconds.

### Bacterial microleakage assessment

**Figure 1:**
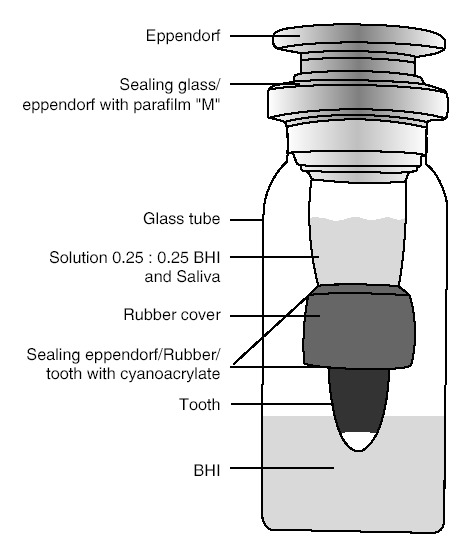
Bacterial microleakage assessment apparatus.

The device assessing the leakage is shown in Figure 1. The teeth were placed inside the 1.5-mL Eppendorf tube. A penicillin vial’s cap was used to secure the connection between the Eppendrof (Elkay, Shrewbury, MA, USA) tube and the tooth used and the connection areas were sealed by cyanoacrylate glue.

The whole system was sterilized by ethylene oxide for 12 hours and then placed in a glass flask containing 4-mm sterile BHI in such a way that 2 mm of the apical root was placed in BHI. The connection areas were sealed by cyanoacrylate glue. For the blindness, the label holding cases and control numbers was attached to the bottom of the vial. To ensure sterilization, the samples were incubated for 1 day at 37^o^C and excluded when discolored by BHI. The rest of the samples were included in the study. Inside the Eppendorf tube of the included samples, 400 µL of BHI (BHI-Oxide LTD, Hanks, USA) suspension containing the bacterial species *Enterococcus faecalis* (0.5 McFarland, ATCC 29212) was added to each microtube and the lids were closed and fresh BHI culture with the bacterium was added to microtubes every 7 days. The samples were then transferred to the incubator. Positive leakage of bacteria was identified as turbidity of culture media. Assessment of turbidity of tubes continued daily for 120 days. Of positive samples, the culture was obtained for proving the existence of colonies of *E. faecalis*.

### Statistical analysis

Data were analyzed using descriptive statistics (means ± standard deviations) and Kaplan Mayer survival analysis with censored data (leakage and non-leakage); log rank test was used to compare different methods using SPSS 18. In this study, P<0.05 was considered significant.

## Results

The survival mean (amount of time without leakage) in PC and MTA is presented in Table 1. There were no significant differences in microleakage between different mixing methods with the use of both materials (P=0.949 for PC and P=0.224 for WMTA). Irrespective of mixing method, the overall survival time of PC was significantly higher than MTA (P=0.003).

**Table 1:** Means and standard deviations for survival time for different mixing methods of WMTA and PC

**Mixing method**	**Material**	**Mean** ^a^	
		**Estimate**	**Std. Error**
**Conventional**	**MTA**	49.133	12.966
	**Portland**	80.267	13.640
**Amalgamator**	**MTA**	66.000	13.327
	**Portland**	78.533	13.462
**Ultrasonic**	**MTA**	69.067	11.505
	**Portland**	84.667	11.416

^a^ Estimation is limited to the largest survival time if it is censored

**Figure 2:**
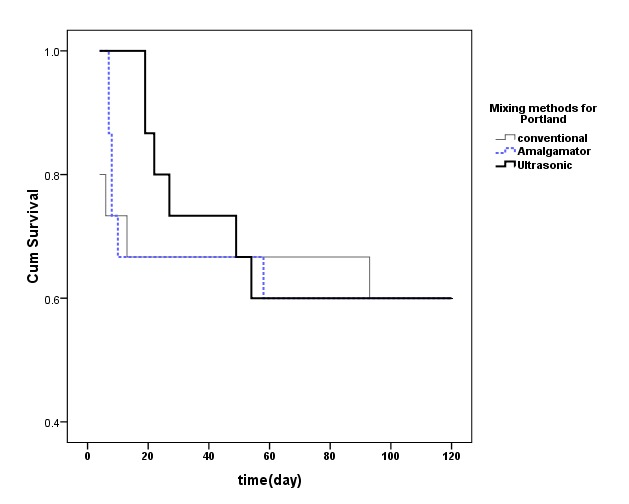
Microleakage survival level of different mixing methods for Portland cement.

**Figure 3:**
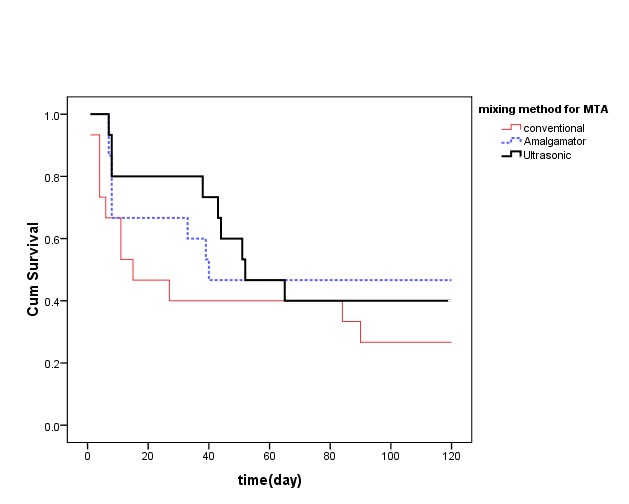
Microleakage survival level of different mixing methods for WMTA.

Microleakage survival level of different mixing methods for PC and WMTA are demonstrated in Figures 2 and 3.

The results of log rank test using SPSS showed:

1. Survival time in the studied mixing methods was not statistically significant for Portland cement (P=0.949).
2. Survival time in the studied mixing methods was not statistically significant for MTA (P=0.224).

## Discussion

The present research aimed to evaluate the effect of different methods of mixing the powder and liquid on the bacterial microleakage of two endodontic biomaterials. The results demonstrated that conventional, amalgamator and ultrasonic mixing methods had no significant effect on bacterial microleakage of the MTA and PC. Moreover, regardless of mixing methods, bacterial microleakage of MTA was more than Portland cement. According to the results, the null hypothesis of the present research was accepted.

Retrograde endodontic treatment is carried out in cases where obtaining the seal between the periodontium and apical foramen is not possible with orthograde treatment. The ability to develop appropriate seal was considered as one of the most important physical characteristics of biomaterials like MTA and PC.^23-26^ It is justifiable due to the technical applications of these materials for root canal treatment because microbial contamination is the main reason for the failure of vital pulp therapies. The periodontal tissue of a tooth that has undergone apical surgery or the apexification method should be free of bacteria and their by-products in order to ensure regeneration.^27,28^

There are several techniques for evaluation of microleakage, including penetration of bacteria or endotoxins, dye penetration, liquid diffusion, protein leakage and the application of radioisotopes. In the present study, bacterial microleakage method was used because it reconstructs the clinical conditions better than other methods and also it is more reliable than dye penetration method.^1^ Different species have been used in several studies in order to assess microleakage. *E. faecalis* was used in the present study as one of the most resistant bacterial species found in endodontic infections; it is found in 24‒77 % of cases. Additionally, it is stored in the laboratory easily and cost effectively.^2,29^

Furthermore in this study, only one bacterial species was used in order to eliminate the effect of confounding factors such as interactions between the bacteria so that interpretation of the results would be easier. In the present research, none of the 5 samples in the negative control group exhibited microbial contamination, which revealed the effectiveness of the present microleakage evaluation. On the other hand, all the samples in the positive control group exhibited microbial contamination, showing that if the root canals remain empty, microbial contamination and leakage will happen in the shortest possible time. This is consistent with the results of previous studies.^1,2,17^ In microbial leakage studies, a number of factors can affect the outcome, including the preparation level of the root canals and the antibacterial effect of the applied materials on the applied bacterial species. In the present study, all of these factors were similar. The length of the root canal, MAF size, and preparation of coronal 1/3 of the canal were selected the same. The thickness of the retrofill materials, which is one of the most effective factors,^3^ was selected the same for all the samples.

Indeed in this study, two areas were questioned, including the microbial leakage of MTA and PC and the effects of different methods of mixing on microleakage. The sealing properties of these materials did not exhibit a difference in the present research, and this result was consistent with the results of a study by Shahi et al on the sealing ability of MTA and PC as the retrofill materials, using dye penetration technique.^17^ However, it was inconsistent with the results of another research by the above researcher in which performance of PC was better with respect to protein leakage in the furcation perforation.^13^ The inconsistency might be attributed to differences in the evaluation methods and differences in the bacterial or and the protein particle size.

The other part of this study was the mixing method because based on previous studies the technique of mixing the hydrophilic cements is an affective factor in the physical properties.^18,19,21,22,26^ In order to achieve the desirable physical and chemical properties, the powder and liquid particles should be connected appropriately, aiming to blend the materials properly. In the present study, the mixing technique had no significant effect on the microbial microleakage in any of the materials. Shahi et al demonstrated in a research that the mixing method had no effect on the push-out bond strength of MTA.^21^ The chemical binding capability of MTA is always considered as one of its excellent characteristics related to the sealing capability of this material. When the powder particles are hydrated, the released calcium hydroxide in the connection with the phosphate of the tissue fluids creates a hydroxyapatite layer, which is the cause of the chemical bonds.^16,30-32^ Since the main chemical composition of MTA and PC is similar,^26^ the expectable bonding mechanism for both materials would be the same. On the other hand, the results of two researches of the above-mentioned researchers would be justifiable with regard to this mechanism.

In microleakage studies, two important issues should be considered. The first is the occurrence of microleakage and the second is the time interval when the leakage occurs.^1^ A long study period will be reasonable for this technique;^33,34^ hence the 120 days was considered in the present research as the study period. In some samples of this research, microleakage was not observed. Therefore a longer interval is suggested for further studies.

Considering the limitations of the present study and the results of previous studies related to the effect of different mixing methods on the physical properties of treated endodontic biomaterial the following items are recommended:

1. Positive characteristics of the amalgamator, and ultrasonic mixing methods would be better to apply in clinical situations because it results in no harm on the sealing capability while it also improves other characteristics like flowability.^22^
2. Considering previous studies, Portland cement can be used in applications similar to MTA.

## Conclusion

Based on the results of this *in vitro* study, we can conclude that different mixing methods do not affect microbial leakage of materials, neither in the white MTA nor in the white Portland cement.

## Acknowledgments

This article is supported by Dental and Periodontal Research Center, Faculty of Dentistry, Tabriz University of Medical Sciences, Tabriz, Iran

## Authors’ contributions

ShahriarShahi: Study design and supervisor •AsmarBashirzadeh: thesis performance • Hamid Reza Yavari: preparation of samples •FarnazJafari: article writing and publication, data analysis •Amin Salem Milani: English edit, sample collection •Negin Ghasemi: article publication advice •Mohammad Samiei: scientific advice

## Funding

None

## Competing interests

The authors declare no competing interests with regards to the authorship and/or publication of this article.

## Ethics approval

As the article was an experimental study, at the time of conducting the study, the proposal was approved in Dental and Periodontal Research Center without ethical committee approval.

## References

[1] Reyhani MF, Ghasemi N, Rahimi S, Milani AS, Barhaghi MH, Azadi A (2015). Apical microleakage of AH Plus and MTA Fillapex(R) sealers in association with immediate and delayed post space preparation: a bacterial leakage study. Minerva Stomatol.

[2] Reyhani MF, Yavari H, Ghasemi N, Rahimi S, Barhaghi MH, Mokhtari H (2015). Comparing the Coronal Seal of Different Thicknesses of MTA with Gutta-Percha after Post Space Preparation. ScientificWorldJournal.

[3] Rahimi S, Shahi S, Nezafati S, Reyhani MF, Shakouie S, Jalili L (2008). In vitro comparison of three different lengths of remaining gutta-percha for establishment of apical seal after post-space preparation. J Oral Sci.

[4] Torabinejad M, Watson TF, Pitt Ford TR (1993). Sealing ability of a mineral trioxide aggregate when used as a root end filling material. J Endod.

[5] Torabinejad M, Chivian N (1999). Clinical applications of mineral trioxide aggregate. J Endod.

[6] Felippe WT, Felippe MC, Rocha MJ (2006). The effect of mineral trioxide aggregate on the apexification and periapical healing of teeth with incomplete root formation. Int Endod J.

[7] Sarkar NK, Caicedo R, Ritwik P, Moiseyeva R, Kawashima I (2005). Physicochemical basis of the biologic properties of mineral trioxide aggregate. J Endod.

[8] Tawil PZ, Duggan DJ, Galicia JC (2015). Mineral trioxide aggregate (MTA): its history, composition, and clinical applications. Compend Contin Educ Dent.

[9] Malhotra N, Agarwal A, Mala K (2013). Mineral trioxide aggregate: part 2 - a review of the material aspects. Compend Contin Educ Dent.

[10] Malhotra N, Agarwal A, Mala K (2013). Mineral trioxide aggregate: a review of physical properties. Compend Contin Educ Dent.

[11] Mohammadi Z, Shalavi S, Soltani MK (2014). Mineral trioxide aggregate (MTA)-like materials: an update review. Compend Contin Educ Dent.

[12] Funteas UR, Wallace JA, Fochtman EW (2003). A comparative analysis of Mineral Trioxide Aggregate and Portland cement. Aust Endod J.

[13] Shahi S, Rahimi S, Hasan M, Shiezadeh V, Abdolrahimi M (2009). Sealing ability of mineral trioxide aggregate and Portland cement for furcal perforation repair: a protein leakage study. J Oral Sci.

[14] Reyes-Carmona JF, Felippe MS, Felippe WT (2010). A phosphate-buffered saline intracanal dressing improves the biomineralization ability of mineral trioxide aggregate apical plugs. J Endod.

[15] Reyes-Carmona JF, Felippe MS, Felippe WT (2010). The biomineralization ability of mineral trioxide aggregate and Portland cement on dentin enhances the push-out strength. J Endod.

[16] Rahimi S, Ghasemi N, Shahi S, Lotfi M, Froughreyhani M, Milani AS (2013). Effect of blood contamination on the retention characteristics of two endodontic biomaterials in simulated furcation perforations. J Endod.

[17] Shahi S, Yavari HR, Rahimi S, Eskandarinezhad M, Shakouei S, Unchi M (2011). Comparison of the sealing ability of mineral trioxide aggregate and Portland cement used as root-end filling materials. J Oral Sci.

[18] Shahi S, Ghasemi N, Rahimi S, Yavari HR, Samiei M, Janani M (2015). The Effect of Different Mixing Methods on the pH and Solubility of Mineral Trioxide Aggregate and Calcium-Enriched Mixture. Iran Endod J.

[19] Basturk FB, Nekoofar MH, Gunday M, Dummer PM (2013). The effect of various mixing and placement techniques on the compressive strength of mineral trioxide aggregate. J Endod.

[20] Basturk FB, Nekoofar MH, Gunday M, Dummer PM (2014). Effect of various mixing and placement techniques on the flexural strength and porosity of mineral trioxide aggregate. J Endod.

[21] Shahi S, Rahimi S, Yavari HR, Samiei M, Janani M, Bahari M (2012). Effects of various mixing techniques on push-out bond strengths of white mineral trioxide aggregate. J Endod.

[22] Shahi S, Ghasemi N, Rahimi S, Yavari HR, Samiei M, Janani M (2015). The effect of different mixing methods on the flow rate and compressive strength of mineral trioxide aggregate and calcium-enriched mixture. Iran Endod J.

[23] Antunes HS, Gominho LF, Andrade-Junior CV, Dessaune-Neto N, Alves FR, Rocas IN (2015). Sealing ability of two root-end filling materials in a bacterial nutrient leakage model. Int Endod J.

[24] de Almeida J, Pimenta AL, Felippe WT (2015). A laboratory assessment of bacterial leakage in MTA apical plugs exposed to phosphate-buffered saline. Acta Odontol Latinoam.

[25] Nanjappa AS, Ponnappa KC, Nanjamma KK, Ponappa MC, Girish S, Nitin A (2015). Sealing ability of three root-end filling materials prepared using an erbium: Yttrium aluminium garnet laser and endosonic tip evaluated by confocal laser scanning microscopy. J Conserv Dent.

[26] Baranwal AK, Paul ML, Mazumdar D, Adhikari HD, Vyavahare NK, Jhajharia K (2015). An ex-vivo comparative study of root-end marginal adaptation using grey mineral trioxide aggregate, white mineral trioxide aggregate, and Portland cement under scanning electron microscopy. J Conserv Dent.

[27] Yun HM, Chang SW, Park KR, Herr L, Kim EC (2015). Combined Effects of Growth Hormone and Mineral Trioxide Aggregate on Growth, Differentiation, and Angiogenesis in Human Dental Pulp Cells. J Endod.

[28] Ghasemi N, Rahimi S, Lotfi M, Solaimanirad J, Shahi S, Shafaie H (2014). Effect of Mineral Trioxide Aggregate, Calcium-Enriched Mixture Cement and Mineral Trioxide Aggregate with Disodium Hydrogen Phosphate on BMP-2 Production. Iran Endod J.

[29] Samiei M, Ghasemi N, Divband B, Balaei E, Hosien Soroush Barhaghi M, Divband A (2015). Antibacterial efficacy of polymer containing nanoparticles in comparison with sodium hypochlorite in infected root canals. Minerva Stomatol.

[30] de Almeida J, Felippe MC, Bortoluzzi EA, Teixeira CS, Felippe WT (2014). Influence of the exposure of MTA with and without calcium chloride to phosphate-buffered saline on the push-out bond strength to dentine. Int Endod J.

[31] Turker SA, Uzunoglu E (2015). Effect of powder-to-water ratio on the push-out bond strength of white mineral trioxide aggregate. Dent Traumatol.

[32] Lotfi M, Ghasemi N, Rahimi S, Bahari M, Vosoughhosseini S, Saghiri MA (2014). Effect of smear layer on the push-out bond strength of two endodontic biomaterials to radicular dentin. Iran Endod J.

[33] Yavari HR, Samiei M, Shahi S, Aghazadeh M, Jafari F, Abdolrahimi M (2012). Microleakage comparison of four dental materials as intra-orifice barriers in endodontically treated teeth. Iran Endod J.

[34] Shahi S, Jeddi Khajeh S, Rahimi S, Yavari HR, Jafari F, Samiei M (2016). Effect of different mixing methods on the bacterial microleakage of calcium-enriched mixture cement. Minerva Stomatol.

